# Genetic variants in the *TORC2* gene promoter and their association with body measurement and carcass quality traits in Qinchuan cattle

**DOI:** 10.1371/journal.pone.0227254

**Published:** 2020-02-14

**Authors:** Rajwali Khan, Sayed Haidar Abbas Raza, Hongfang Guo, Wang Xiaoyu, Wu Sen, Syed Muhammad Suhail, Abdur Rahman, Irfan Ullah, Ayman Hassan Abd El-Aziz, Zeinab Manzari, Akil Alshawi, Linsen Zan

**Affiliations:** 1 College of Animal Science and Technology, Northwest A&F University, Yangling, Shaanxi, P.R. China; 2 Qinghai Academy of Animal Science and Veterinary Medicine, Qinghai University, Xining, China; 3 Department of Livestock Management, Breeding and Genetics, The University of Agriculture Peshawar, Peshawar, Pakistan; 4 College of Bio-medical Engineering, Chongqing University, Chongqing, China; 5 Animal Husbandry and Animal Wealth Development Department, Faculty of Veterinary Medicine, Damanhour University, Damanhour, Egypt; 6 Department of Animal Science, College of Agriculture and Natural Resources, University of Tehran, Karaj, Iran; 7 School of Life Science University of Nottingham, Nottingham, United Kingdom; 8 National Beef Cattle Improvement Research Center, Yangling, China; Universita degli Studi di Bologna, ITALY

## Abstract

The *TORC2* gene is responsible for nutrient metabolism, gluconeogenesis, myogenesis and adipogenesis through the PI3K-Akt, AMPK, glucagon and insulin resistance signaling pathways. Sequencing of PCR amplicons explored three novel SNPs at loci g.16534694G>A, g.16535011C>T, and g.16535044A>T in the promoter region of the *TORC2* gene in the Qinchuan breed of cattle. Allelic and genotypic frequencies of these SNPs deviated from Hardy-Weinberg equilibrium (HWE) (*P* < 0.05). SNP1 genotype GG, SNP2 genotype CT and SNP3 genotype AT showed significantly (P <0.05) larger body measurement and improved carcass quality traits. Haplotype H1 (GCA) showed significantly (p<0.01) higher transcriptional activity (51.44%) followed by H4 (ATT) (34.13%) in bovine preadipocytes. The diplotypes HI-H3 (GG-CC-AT), H1-H2 (GG-CT-AT) and H3-H4 (GA-CT-TT) showed significant (*P*<0.01) associations with body measurement and improved carcass quality traits. Analysis of the relative mRNA expression level of the *TORC2* gene in different tissues within two different age groups revealed a significant increase (*P*<0.01) in liver, small intestine, muscle and fat tissues with growth from calf stage to adult stage. We can conclude that variants mapped within *TORC2* can be used in marker-assisted selection for carcass quality and body measurement traits in breed improvement programs of Qinchuan cattle.

## 1. Introduction

There are three members of TORC gene family: *TORC1*, *TORC2* and *TORC3*, which is also known as CRTC [CREB (cAMP response element binding protein)-regulated transcription coactivator]. The *TORC2* gene is responsible for nutrient metabolism, gluconeogenesis, myogenesis and adipogenesis through the PI3K-Akt, AMPK, glucagon and insulin resistance signaling pathways via promotion of the anabolic and inhibiting catabolic processes within the cells [1]. Moreover, *TORC2* is a coactivator gene that plays a key role in glucagon-mediated activation of gluconeogenesis through a synchronized mechanism of glucocorticoid receptor and glucagon-CREB pathways coordinated with *PEPCK* and *G6P* genes [2–6]. Additionally, through the CREB pathway in coordination with *peroxisome proliferator-activated receptor γ* (*PPARγ*), *coactivator 1α (PGC1α)* and *NR4A (nuclear receptor subfamily 4 group A)*, *CRTC2* promotes gluconeogenesis, adipogenesis [7] and myogenesis [8]. In adipose tissue, *TORC2* plays a role in the anabolic processes of adipocytes in lipid metabolism, including lipogenesis, adipogenesis and lipid esterification [9, 10]. *TORC2* is one of the main inhibitors of lipolysis by its regulation of *PKA* and *HSL* activities [11]. Moreover, all members of the *TORC* gene family regulated cell proliferation through *PII* activity in human preadipocytes [12]. *TORC2* modulates triglyceride synthesis and lipogenesis through regulation of *SREBP1*, which in turn regulates other fat-related genes [13]. Additionally, the CREB pathway also performs an important function in skeletal muscle. Transgenic mice with A-CREB negative gene exhibited skeletal muscle dystrophy characterized by muscular wasting, muscular inflammation and myonecrosis [14]. The above findings confirmed the role of *TORC2* in adipocyte and myocyte proliferation and differentiation. Therefore, investigation of *TORC2* genetic polymorphisms might contribute to breed improvement programs for carcass quality and body measurement traits in beef cattle. Body measurement and carcass quality traits are used for the assessment of animals’ production. The loin area muscle and intramuscular fat contents are the key indicators of meat quality grading. These traits are mostly affected by the age of the animals, management conditions such as nutrition and by the genetics of the animals [15, 16]. To achieve sustainable improvement in these traits of economic importance, selective breeding is an effective strategy, but it takes a very long time to obtain efficient genetic gain due to the longer generation interval in cattle. Genomic selection increases the rate of genetic improvement and reduces cost of progeny testing by allowing breeders to preselect animals that inherited chromosome segments of greater merit [17, 18]. Single nucleotide polymorphism (SNP) markers can now cover the genome with high density and are inexpensive to obtain. Evaluations based on SNP genotypes can be computed as soon as DNA can be obtained, which allows selection in both sexes early in life [19, 20]. Causal variants identification increase the accuracy of genomic selection, which is considered an efficient way of analyzing the association between genetic polymorphism and traits of economic importance [21].

Polymorphism in the promoter region is of great importance because the promoter region contains cis-acting elements that bind with transcription factors and regulate the respective gene [22–25]. For example, single nucleotide polymorphisms in the *STAT3* [23], *SIRT3* [26], *KLF3* [27] *SIX1* [24] and *SIX4* [28] genes promoter regions impacted body measurement and meat quality traits in the Qinchuan cattle breed. Polymorphisms in the promoter region cause the addition or loss of important transcription factor binding sites, which in turn leads to the suppression or activation of target genes. We hypothesized that variants in the promoter region of *TORC2* gene may influence phenotype, especially body measurement and carcass quality traits in Qinchuan cattle. Therefore, this study was designed with the objective of exploring SNPs in the *TORC2* gene promoter region, the impact of these SNPs on the sequences of transcription factor binding sites and their association with body measurement and carcass quality traits.

Hence, the findings of the present study will enhance our understanding of the *TORC2* gene regulation pattern, and the genetic variants may contribute to marker-assisted selection for breed improvement programs of Qinchuan cattle.

## 2. Materials and methods

### 2.1 Ethical statement

The China Council on Animal Care guidelines were used when dealing with animals throughout the experiments. Approval was also granted for all the experimental protocols by the Experimental Animal Management Committee (EAMC) of Northwest Agriculture and Forestry University, Yangling, China. For the approved protocols that were used when dealing with animals notified vide Notification No. EAMC/20-23 dated 20.04.2013.

### 2.2 Phenotypic data and DNA sample collection

In total, 428 female cows aged 18 to 24 months were randomly selected from the National Beef Cattle Improvement Center’s experimental farm (Yangling, China). The subject animals were fed a total mixed ration (TMR) containing 25% concentrate and 75% roughages of dry straw and corn silage, and water was offered ad libitum. The feeding was offered based on NRC standards (Nutrient Requirement of Beef Cattle) and in the similar rearing environment (similar temperature, humidly etc.) [29]. Carcass quality traits, including IF% and ULA, were estimated as per standard procedure [26, 30] using ultrasound technology (Sono-grader ultrasound machine, Renco, USA). The carcass quality traits were estimated in live animals according to the manufacturer’s instructions. The ultrasonic probe was placed between the 12 and 13th rib area, and the carcass quality traits including ultrasound loin area (ULA) and intramuscular fat percentage (IF%) were recorded. The blood samples were also collected from these animals and transferred to the molecular laboratory of National Beef Cattle Improvement and Research for DNA extraction. The phenol chloroform protocol was used for the extraction of genomic DNA from the collected blood samples [31].

### 2.3 PCR amplification and genotyping

Primers (Table 1) were designed using Primer Premier 5 software (PREMIER Biosoft International, CA, USA) for the amplification of the -630 bp promoter region, upstream of the transcription start site of the bovine *TORC2* gene GenBank NC_037330.1. The KOD plus Neo Enzyme Kit (TOYOBA, Japan) was used for PCR amplification according to the manufacturer’s instructions. Genomic DNA from 428 Qinchuan cattle was used as a template for PCR amplification. Thermocycling (PCR) was performed using 3-step cycle conditions with pre-denaturation temperature at 94.0°C for 5 minutes followed by 34 cycles of denaturation temperature at 97.0°C for 30 seconds, annealing Tm of the primers used (see Table 1)°C for 30 seconds and final extension temperature at 72.0°C for 45 seconds. The PCR products were sequenced through Sangon (Shanghai, China) to screen for polymorphisms. All sequences were checked using Seq Man (DNASTAR, Inc., USA) software, and the SNPs were identified.

**Table 1:** Primers used for the amplification and expression of *TORC2* gene.

Name	Purpose	Primer sequence	Tm (^0^C)	Product length (bp)
Forward *TORC2*	SNPs detection	TGGGATACAGCTGGGGATCA	58.18	945
Reverse *TORC2*		TCCAGCTTTAGGGCACACTG	57.71	
Forward *TORC2*	Gene expression	GAGGAGGTGATGATGGAC	52.05	138
Reverse *TORC2*		GCTCTGGAACTCGGCTAG	55.57	
Forward GAPDH	Housekeeping gene	AGTTCAACGGCACAGTCAAGG	58.09	124
Reverse GAPDH		ACCACATACTCAGCACCAGCA	58.98	
Forward β- Actin	Housekeeping gene	ATCGGCAATGAGCGGTTC	60.00	144
Reverse β- Actin		CGTGTTGGCGTAGAGGTC	60.00	

### 2. 4 Potential cis-acting element identification

Genomatix Mathinspector software (http://www.genomatrix.com) was used for the identification of the transcription factor (TF) binding sites in the promoter region of the bovine *TORC2* gene with the cut off value of 90%. The mutations in the sequences of respective transcription factor binding sites due to SNPs were also checked through the same Genomatix online suite for their specific position and significance (Ci value), and the important transcription factor binding sites that were altered due to respective SNPs were identified. The ci-vector (consensus index vector) value of the respective cis-acting element indicate significance of the mutated nucleotides (SNPs) in the sequences of the respective transcription factor binding sites [32].

### 2.5 Construction of plasmid, isolation, culture and transfection of preadipocyte cells for luciferase reporter assay

The roles of TF binding sites, which are affected by the SNPs in the *TORC2* gene promoter, were assessed through a dual luciferase activity assay. The four selected haplotypes were amplified using specific primers with added enzyme site sequences of *Sac I* and *Hind III* enzymes to forward and reverse primers, respectively (Table 1). The PCR amplicons were cloned into pMD-19 T-Vector (Takara, Japan) and digested with *Sac I* and *Hind III* restriction enzymes (Takara, Japan). The haplotype DNA was then extracted from the gel through the E.Z.N.A gel extraction kit (Omega, Biotek, Inc, USA) and ligated through T4 ligation (Takara, Japan) into pGL3 basic (luciferase reporter vector), which was also digested with *Sac I* and *Hind III* (Takara, Japan) restriction enzymes. The bovine preadipocyte cells were collected from healthy newborn calves (5 days old) of Qinchuan cattle breed at the experimental farm of National Beef Cattle Improvement Center of Northwest A&F University, located in Yangling, Shaanxi, China [33]. The cells were cultured and maintained in HyClone^™^ Dulbecco’s Modified Eagle’s Medium (DMEM)/F12 1:1 cell culture media (ThermoFisher Scientific, Inc. USA), supplemented with 10% FBS (fetal bovine serum) and 1% antibiotic (penicillin 100 IU/mL and streptomycin 100 μg/mL) in an atmospheric incubator at 37°C and 5% CO_2_. The cells were plated in 24-well plates and transiently transfected at 70–90% confluence with Lipofectamine 3000 (Invitrogen, USA), and 10 ng of pRL-TK was used as a normalizing reporter vector along with 500 ng of luciferase reporter vector (pGL3-basic) harboring DNA of selected haplotypes. Forty-eight hours post-transfection, the cells were lysed, and both firefly luciferase and Renilla luciferase activities were measured as per the standard protocol of the dual luciferase reporter assay (Promega, USA) using a Nano Quant Plate TM (TECAN, Infinite, M200 PRO System).

### 2.6 Estimates of conservation and biological evolution

**Fig 1:**
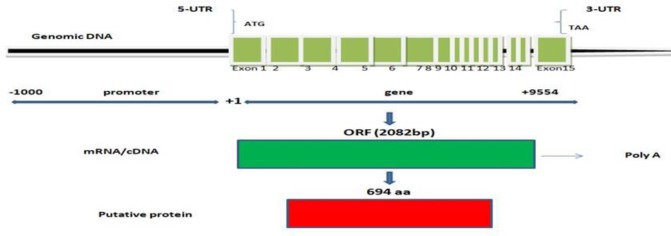
Structure of *TORC2* gene, the source of information was (https://www.ncbi.nlm.nih.gov/gene/540959).

The *TORC2* gene is located on chromosome 3 of the bovine genome. The total length of *TORC2* is 9,554 bp, comprising the genomic coordinates starting from 16,477,661 to 16,487,214 (NC_037330.1, Reference genome *bos taurus* ARS-UCD1.2). This gene comprises 15 exons, the ORF which started from the start codon to the stop codon is 2082 bp, and the putative protein contains 694 amino acids (Fig 1). The sequences of amino acids of the *TORC2* gene were obtained from the NCBI search for the following 10 species (*Bos taurus* NP_001069718.1; *Bos indicus* XP_019810560.1; *Bubalus bubalis* XP_006044208.1; *Ovis aries musimon* XP_011987586.1; *Capra hircus*; XP_017901639.1; *Homo sapiens* NP_859066.1; *Mus musculus* NP_001344081.1; *Canis lupus familiaris* XP_013970885.1; *Sus scrofa* P_005663462.1; and *Gallus gallus* XP_015154073.1) (www.ncbi.nlm.nih.gov/protein). The sequences were downloaded through TBtools (toolbox for biologists) v.0.58 software in fasta format. Protein sequences of all ten (10) species (multiple sequence alignment) were aligned through MUSCLE sequencing alignment (multiple sequence comparison through log expectation), and the phylogenetic tree (neighbor-joining) was built. MEGA version 7.0.26 (Philadelphia, PA, USA) software was used for both multiple sequence alignment and phylogenetic tree construction [34]. For the analysis of protein structure and function, the motifs were searched, and conserved domains were identified through the online MEME suite website [35], CDD NCBI and TBtools [36, 37].

### 2.7 Tissue collection, RNA extraction, preparation of cDNA and real-time PCR

For tissue collection, three samples from Qinchuan calves (7 days old) and three samples from mature Qinchuan cattle (two years old) were selected. The animals from the two groups (calf and mature) were nonrelatives for at least three generations. The animals were dressed in a local abattoir under standard procedure of animal stunning, exsanguination and skinning. To measure the relative expression of the *TORC2* gene, eight tissues, including dorsal muscle, fat, heart, kidney, lung, liver, rumen and small intestine, were collected from both the calf and mature Qinchuan groups. After collection from the carcass, the tissue samples were preserved in liquid nitrogen and were transferred immediately in frozen form to the molecular laboratory for the extraction of total RNA. Total RNA was extracted from the tissue using TRIzol^™^ reagent (Invitrogen, ThermoFisher Scientific, Inc. USA) and subjected to reverse transcription for synthesis of cDNA using the PrimeScriptTM RT Reagent Kit with gDNA eraser (Perfect Real Time, Takara). Quantitative real time (RT-PCR) was performed using the Sybr Premix EX Taq Kit (Takara, Dalian, China). The prepared cDNA of each tissue was used as a template, and gene-specific primers (Table 1) were used in a 20 μL reaction mixture. Two bovine genes, *GAPDH (glyceraldehyde-3-phosphate dehydrogenase)* and *β-actin*, from NCBI website, with mRNA GenBank accession numbers NM_001034034.2 and NM_173979.3, respectively, were used as endogenous control genes. The thermocyclic reaction was performed using a thermocycler 7500 system SDS V 1.4.0 (Applied Biosystems, USA), with cyclic reaction conditions of preheating at 95°C for 5 minutes, a total of 34 cycles of denaturation temperature at 95°C for 30 seconds, annealing temperature at 60°C for 30 seconds and extension temperature at 72°C for 30 seconds. The thermocylic reactions were run in triplicate for each tissue sample, and the mRNA relative expression levels were calculated using the 2^−ΔΔCt^ method [38].

### 2.8 Data analyses

The general linear model (GLM) using SPSS 20.0 software (SPSS, Inc., Chicago, USA) was used for the association analysis between SNPs and selected traits of carcass quality. The linear model was the same as that used in previous publications of our research group [23, 39, 40]. The statistical linear model was Y_ijkm_ = u+G_i_ = A_j_+A_k_+S_m_+E_ijkm_, where *Y*_*ijkm*_ are the traits measured on each animal; *u* is the overall mean for each trait; *G*_*i*_ is the fixed effect associated with *j*th genotype; *A*_*j*_ is the fixed effect of the *j*th age; *A*_*k*_ is the fixed effect due to the age of the dam; *S*_*m*_ is the random effect with the *m*th sire; and E_ijkm_ is the standard error.

A similar statistical linear model to the model above with slight modifications was used for the association of combined genotypes; in this analysis, *G*_*j*_ represents the fixed effect associated with the *j*th combined genotypes. The Bonferroni correction was made for the adjustment of the p values.

The allelic and genotypic frequencies for all three SNPs were calculated. Estimation of Hardy-Weinberg equilibrium (HWE) was measured through the chi square test in Pop Gene software version 3.2 [41]. Population genetic indicators, such as gene heterozygosity (He) and polymorphism information content (PIC), were measured through established methods [42]. The haplotypes and the D’ and r^2^ linkage disequilibrium (LD) were determined through Haploview (http://analysis.bio-.cn/myAnalysis.php) [43]. GraphPad Prism (6.0) was used to perform Dunnett’s multiple comparisons test for the analysis of dual luciferase reporter transcriptional activities of selected haplotypes normalized against the activity of blank luciferase reporter vector.

The *TORC2* gene mRNA relative expression levels were calculated using the 2^−ΔΔCt^ method [38]. The data were expressed as the means ± SE, and p<0.05 was considered an accepted value for statistical significance.

## 3. Results

### 3.1 SNP identification

Three SNPS at loci g.16534694G>A, g.16535011C>T and g.16535044A>T in the promoter region of the *TORC2* gene were identified. SNP1 yielded two genotypes, including GA and GG, while SNP2 produced CC, CT and TT. The genotypes produced by SNP3 were AA, AT and TT (Table 2). Allelic and genotypic frequencies analysis exhibited that all three SNPs deviated from the Hardy-Weinberg equilibrium (HWE) (Table 2, p < 0.05). In the present study, SNP1 showed a low PIC value, while the PIC classification of SNP2 and SNP3 were found to be moderately polymorphic (0.25 < PIC < 0.50) [44].

**Table 2:** Genotype frequencies (%) of *TORC2* gene SNPs in the Qinchuan cattle population.

Site	Genotypic frequencies			Total	Allelic frequencies		HWE	PIC	He	Ne
**SNP1G>A**	GG	GA	AA	428	G	A	5.126	0.173	0.192	1.237
	0.785	0.215	0.000		0.893	0.107				
**SNP2C>T**	CC	CT	TT	428	C	T	3.720	0.345	0.443	1.794
	0.470	0.400	0.130		0.669	0.331				
**SNP3A>T**	AA	AT	TT	428	G	T	0.890	0.374	0.499	1.995
	0.287	0.474	0.238		0.525	0.475				

HWE Hardy- Weinberg equilibrium; X0.052 = 5.991 and X0.012 = 9.210; He denotes gene heterozygosity; Ne denotes effective allele numbers and PIC stands for Polymorphism information contents

### 3.2 Linkage disequilibrium and haplotype identification of the bovine *TORC2* gene

The LD (D'/γ^2^) was highest between SNP2 and SNP3 (0.856/0.399). The LD between SNP1 and SNP2 was 0.748/0.136, while SNP1 and SNP3 was 0.843/0.095. Eight haplotypes were found, but four haplotypes with a frequency of <5% were excluded, and the frequencies of the remaining (four) haplotypes are presented in Table 3.

**Table 3:** Bovine TORC2 gene haplotypes and their frequencies in Qinchuan cattle.

Haplotype	g.SNP1G>A	g.SNP2C>T	g.SNP3A>T	Frequency (%)
Hap1	G	C	A	0.485
Hap2	G	T	T	0.216
Hap3	G	C	T	0.161
Hap4	A	T	T	0.081

The remaining (04) haplotypes with frequency <0.05 were omitted, hence sum of the frequencies of these four selected haplotypes is not equal to 1.0

### 3.3 Association of genotype and diplotype with physical measurements and carcass quality traits

Three SNPs located in the promoter region of the bovine *TORC2* gene exhibited associations with body measurement and carcass quality traits of economic importance in the Qinchuan breed of cattle (Table 4). In SNP1, the cattle with genotype GG showed significantly (P <0.01 and P <0.05) larger body length (BL), hip height (HH), chest depth (CD), chest circumference (CC), ultrasound loin area (ULA) and intramuscular fat percentage (IF%) than genotype GA, while no significant variation was found in the hip width (HW) body measurement trait in both genotypes of SNP1. In SNP2, the cattle with genotype CT exhibited significantly (P< 0.01 and 0.05) larger body length (BL), hip width (HW), chest depth (CD), chest circumference (CC) and intramuscular fat percentage (IF%) than genotypes CC and TT, while no significant variation was found in hip height (HH) and ultrasound loin area (ULA) traits in all three genotypes of SNP2. In SNP3, the cattle with genotype AT showed significantly (P< 0.01 and P< 0.05) larger body length (BL), chest depth (CD) and ultrasound loin area (ULA) than genotypes AA and TT.

**Table 4:** Genotype association of *TORC2* gene with body measurement and carcass quality traits of Qinchuan cattle.

Locus	Genotype	BL(cm)	HH(cm)	HW(cm)	CD(cm)	CC(cm)	ULA(cm^2^)	IF(%)
SNP1 (g.16534694)	GA(92)	132.054±0.272	121.880±0.392	38.467±0.469	57.005±0.336	160.109±0.531	44.167±0.448	7.001±0.124
	GG(336)	133.957±0.216	122.894±0.205	38.973±0.246	59.025±0.266	162.693±0.391	46.048±0.429	7.998±0.123
	**P-*value***	**P<0.01**	**P<0.05**	**NS**	**P<0.05**	**P<0.01**	**P<0.01**	**P<0.05**
SNP2 (g.16535011)	CC(201)	133.393±0.273	122.410±0.266	38.537±0.316	58.672±0.343	161.353±0.361	45.839±0.308	7.122±0.083
	CT(171)	134.082±0.536	123.111±0.289	39.503±0.343	59.383±0.372	163.333±0.278	45.443±0.334	7.482±0.090
	TT(56)	132.473±0.338	122.304±0.504	38.089±0.599	57.527±0.650	161.304±0.684	45.560±0.584	7.462±0.065
	**P-*value***	**P<0.05**	**NS**	**P<0.05**	**P<0.05**	**P<0.01**	**NS**	**P<0.01**
SNP3 (g.16535044)	AA(117)	132.308±0.363	122.368±0.350	39.034±0.417	58.560±0.449	161.179±0.478	45.229±0.401	7.206±0.110
	AT(209)	134.521±0.414	122.818±0.262	38.871±0.312	59.392±0.509	162.627±0.358	46.226±0.300	7.279±0.082
	TT(102)	132.975±0.389	122.740±0.375	38.657±0.447	57.887±0.481	162.235±0.512	44.928±0.234	7.494±0.118
	**P-*value***	**P<0.01**	**NS**	**NS**	**P<0.05**	**P<0.05**	**P<0.01**	**NS**

The bonferroni correction was used for the adjustment of p values. BL (Body length); HH (Hip height); HW (Hip width); CD (Chest depth); CC (Chest circumference); ULA (Ultrasound loin area) and IF% (intramuscular fat percentage)

These four haplotypes produced five diplotypes (haplotype combinations), which showed significant (P< 0.01 and P< 0.05) associations with body measurement and carcass quality traits (Table 5). Qinchuan cattle with diplotype HI-H3 (GG-CC-AT) showed significantly (p<0.01) larger body length (BL) and ultrasound loin area (ULA). Cattle with diplotype H1-H2 (GG-CT-AT) exhibited significantly larger chest depth (CD) and chest circumference (CC). Significantly (P<0.05) larger hip width and intramuscular fat percentage (IF%) were found in cattle having diplotype H3-H4 (GA-CT-TT).

**Table 5:** Association of diplotype combination of *TORC2* gene with body measurement and carcass quality traits of Qinchuan cattle.

Diplotype (Nos)	BL(cm)	HH(cm)	HW(cm)	CD(cm)	CC(cm)	ULA(cm^2^)	IF (%)
H1-H1(GG-CC-AA) (104)	132.135±0.382	122.159±0.372	38.846±0.427	58.356±0.464	161.642±0.566	44.996±0.413	7.162±0.225
H1-H3(GG-CC-AT) (81)	135.191±0.433	122.735±0.421	37.926±0.401	59.220±0.436	161.464±0.963	47.253±0.468	6.960±0.132
H1-H2(GG-CT-AT) (118)	134.000±0.359	122.873±0.349	39.536±0.484	59.644±0.526	163.051±0.469	45.706±0.388	7.432±0.110
H3-H4(GA-CT-TT) (28)	134.018±0.736	123.268±0.716	39.585±0.823	58.286±0.894	161.058±0.500	45.050±0.796	7.604±0.117
H2-H4(GA-TT-TT) (41)	132.134±0.608	122.073±0.592	37.805±0.680	57.220±0.739	161.073±0.796	45.455±0.658	7.459±0.186
*P value*	**P<0.01**	**NS**	**P<0.05**	**P<0.05**	**P<0.05**	**P<0.01**	**P<0.05**

### 3.4 Transcription factor binding site prediction

In silico analysis showed that mutations due to SNPs caused gains and loss of transcription factor binding sites (Table 6 and Table 7). The G> A in SNP1 caused the loss of three important transcription factor binding sites, E2F7, PRDI and ARP1. In SNP2, the T>C also caused the loss of three important transcription factor binding sites (AREB6, FOXP1, and NFAT). However, none of the cis-acting element binding sites were changed due to SNP3 (Table 7). Similarly, with G>A in SNP1, six (6) new potential cis-acting element binding sites (KLF6, KLF1, MAZ, HSF2, MZF1, and SPI1) were gained, while in SNP2, the C>T causes a gain of one transcription factor binding site KLF2 (Table 7).

**Table 6:** Loss of transcription factors binding sites due to changes in promoter sequence through SNP1 (G to A) and SNP2 (T to C).

SNP	Variation loci	Name of TF	TF Binding site Sequence	Strand	Score*	ci-value
**SNP1**	g -618 G>A	E2F transcription factor 7 (E2F7.02)	ggggggaGGGAaaggct	+	90.9	>60
		PRDI (positive regulatory domain I element) binding factor 1(PRDM1.02)	ggggaggGAAAggctcacc	+	84.1	>60
		Nuclear receptor subfamily 2 factors (ARP1.01)	ggggggagggaaagGCTCaccccca	+	84.0	>60
**SNP2**	g -300 C>T	Two-handed zinc finger homeodomain transcription factors (AREB6.04)	tccctGTTTccac	+	99.1	>60
		Fork head domain factors (FOXP1.01)	ggggtggAAACagggag	-	94.0	>60
		Nuclear factor of activated T-cells (NFAT.01)	aggggtGGAAacagggagg	-	97.4	>60

*Core Similarity score; red colored alphabets reflect the core sequence nucleotides within the TF binding site; encircled nucleotide represents mutated nucleotide

**Table 7:** Gain of new transcription factors binding sites due to changes in promoter sequence through SNP 1(A to G) and in SNP2 (C to T).

SNP	Variation loci	Name of TF	TF Binding Sequence	Strand	Score	ci-value
**SNP1**	g -618 G>A	Krueppel like transcription factors (KLF6.01)	caagggGGGGaggggaagg	+	95.7	<60
		Krueppel like transcription factors (KLF.02)	agggggGGAGgggaaggct	+	98.9	<60
		Myc associated zinc fingers (MAZ.01)	ggggGAGGggaag	+	97.0	>60
		Heat shock factors (HSF2.01)	aagggggggagggGAAGgctcaccc	+	88.1	>60
		Myeloid zinc finger 1 factors (MZF1.02)	gaGGGGaaggc	+	100	>60
		Human and murine ETS1 factors (SPI1.02)	gggggaggGGAAggctcaccc	+	96.5	>60
**SNP2**	g -300 C>T	Krueppel like transcription factors (KLF2.01)	ggaagGGGTggagacaggg	-	100	>60

### 3.5 Luciferase reporter assay

**Fig 2:**
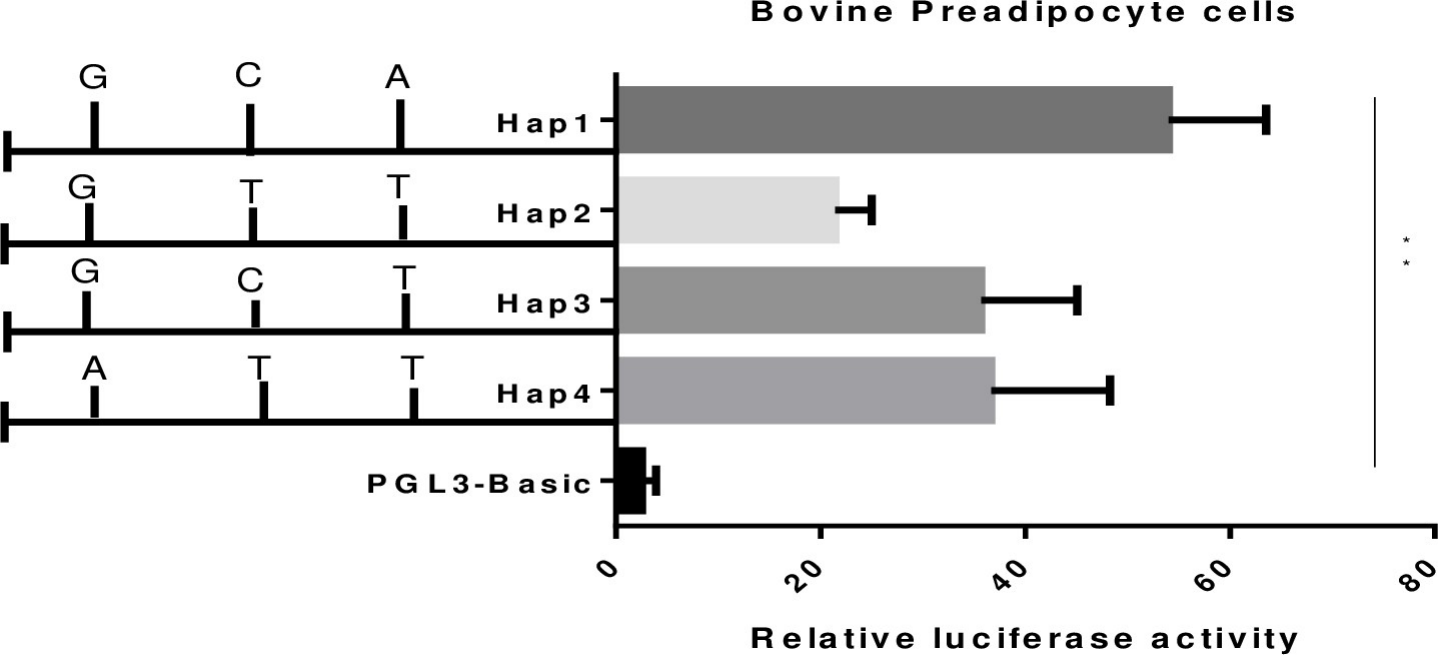
Transcriptional activities of haplotypes reported as relative luciferase activity using dual luciferase reporter assay in bovine preadipocyte cells. Significant variation (p<0.01) of the Firefly luciferase activity normalized against Renilla luciferase activity of respective haplotypes are shown as (**).

To determine the transcriptional activities of different haplotypes, a dual luciferase reporter assay was conducted. The results analyzed with Dunnett’s multiple comparisons test showed significant (p<0.01) variation in the transcription activities of these selected haplotypes (Fig 2). The mean difference in the transcriptional activity of H1 was 51.44 (p<0.01) followed by H4 (34.13) (p<0.01) compared to the transcription activity of the pGL3-Basic vector.

### 3.6 Bioinformatics study of the *TORC2* gene

**Fig 3:**
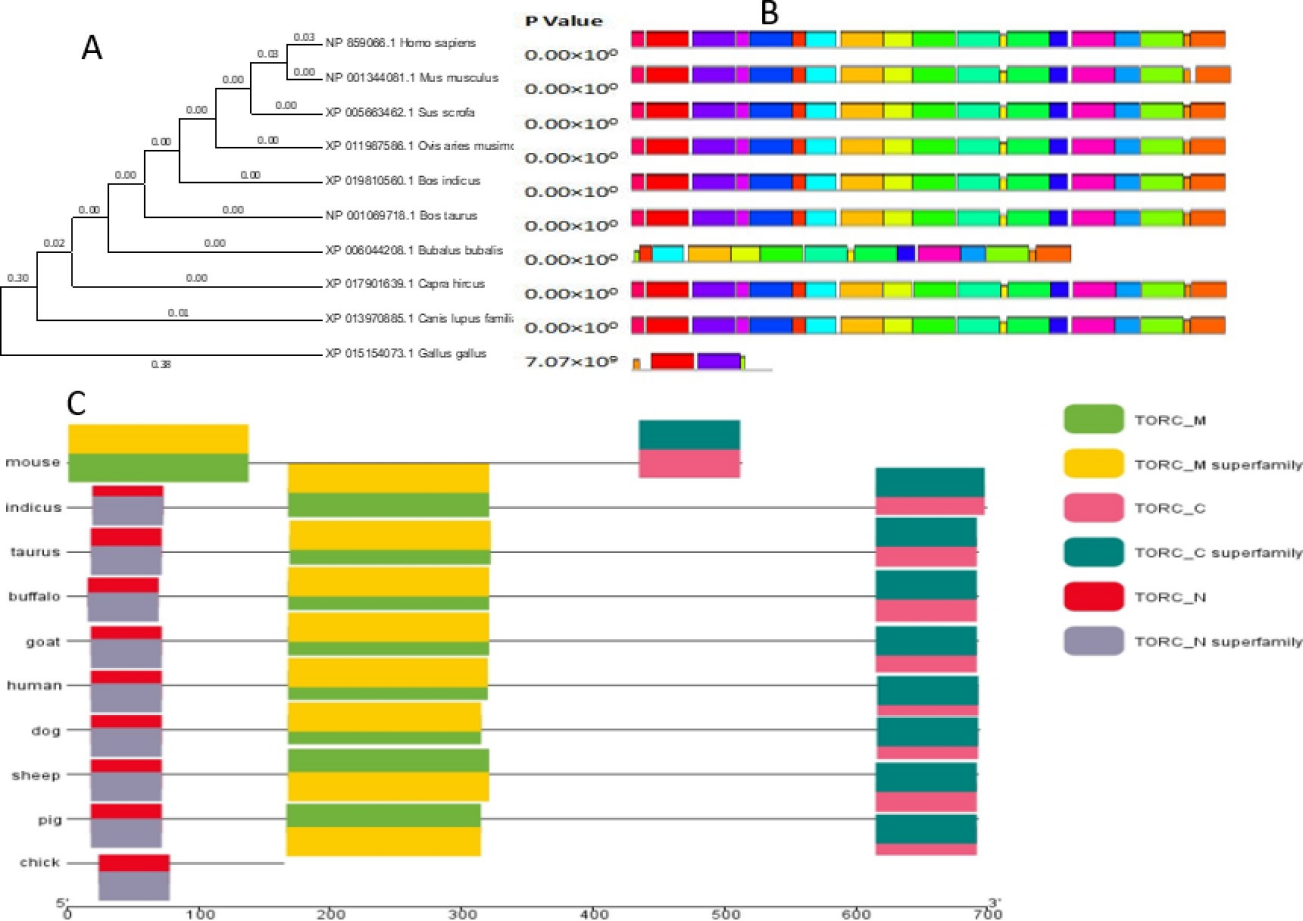
The Phyolgenetic tree (A), conserved structural motifs (B) of ten species. The p- value shows the significance of the motif site. The length of the color block shows the position, strength and significance of a particular motif site. The motif sites length is proportional to the negative logarithm of the p-value of the motif site. These colors are given through motif analysis performed through MEME suit system. (C) Conserved domain families of TORC2 protein in ten different species represented by different color blocks, reflects specific hit of different domain family in different species. Twenty significant TORC2 protein motifs within 10 different selected species, identified through MEME suit. The different colors within the motifs represent abbreviation of different amino acids.

**Fig 4:**
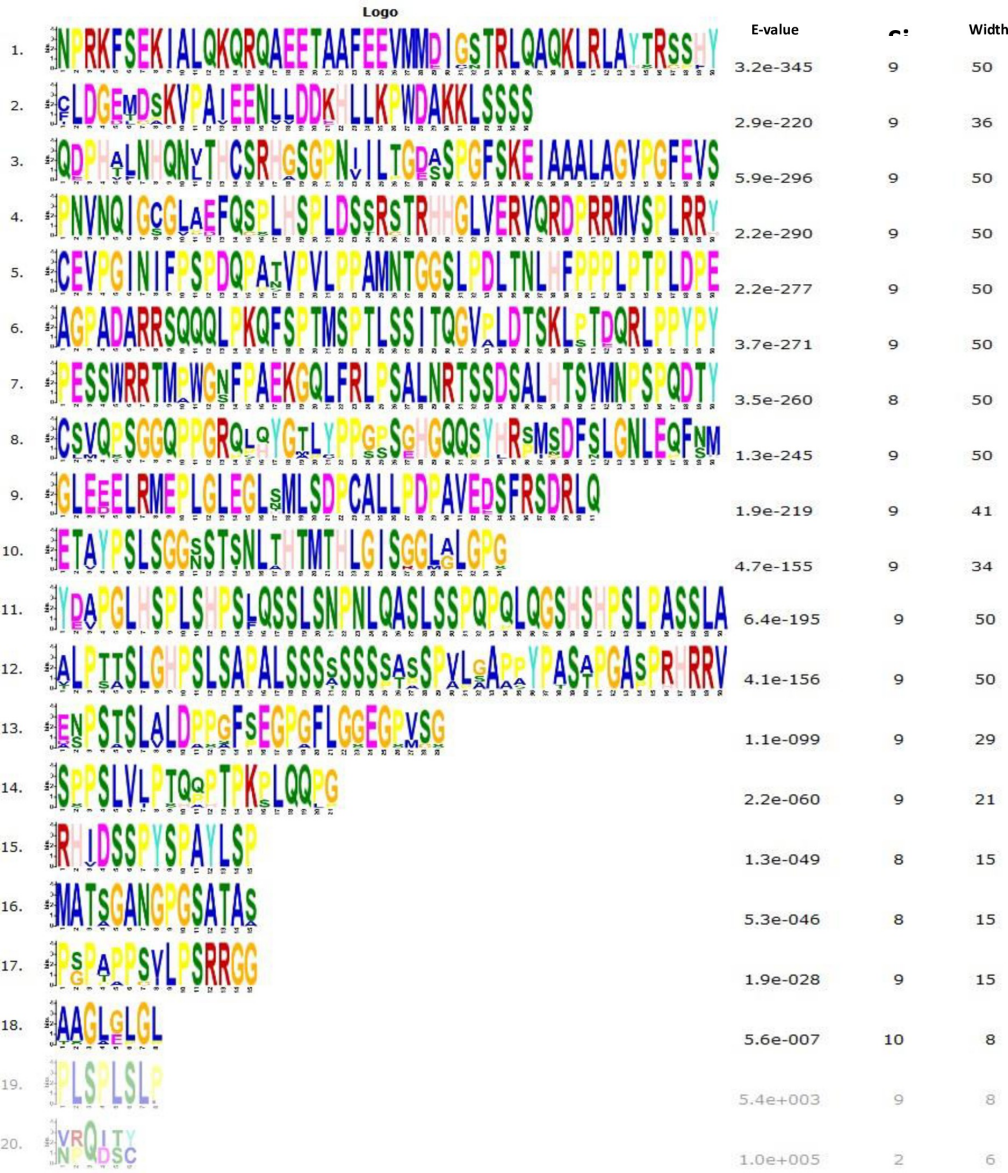
Twenty significant TORC2 protein motifs within 10 different selected species, identified through MEME suit. The different colors within the motifs represent abbreviation of different amino acids.

The multiple sequence alignment of the TORC2 protein was performed for 10 species (S1 Fig). The protein structure was highly conserved among the 7 mammalian species but was up to 40% different in *Mus musculus*, *Canis lupus familiaris*, and *Gallus gallus*. A phylogenetic tree was constructed using MEGA 7 software (Fig 3A). The MEME online suit was used to find common significant motifs in the super secondary protein structure of the *TORC2* gene in 10 target species (Fig 3B). *Bos indicus*, *Bubalus bubalis*, *Ovis aries musimon* and *Capra hircus* were the most closely related species with *Bos taurus*. *Sus scrofa*, *Mus musculus*, *Canis lupus familiaris* and *Homo sapiens* species branches were found far away from the *Bos taurus* sequence. Protein tertiary structures of the *TORC2* gene were searched for all the tested species through NCBI CDD (Conserved Domains Database) and found 6 specific conserved domain hits of super families (TORC_M, TORC_ super family, TORC_C, TORC_ super family, TORC_N and TORC_N super family) in tested species (Fig 3C). The two domains hits (TORC_N and TORC_N super family) were not conserved in *Mus musculus*. For the rest of the species, all domains hits were conserved. A total of 20 significant motifs were found among 10 species (Fig 4), which indicated functional similarity among the selected species at the protein super secondary structure level.

### 3.7 Relative mRNA expression of the *TORC2* gene at different ages

**Fig 5:**
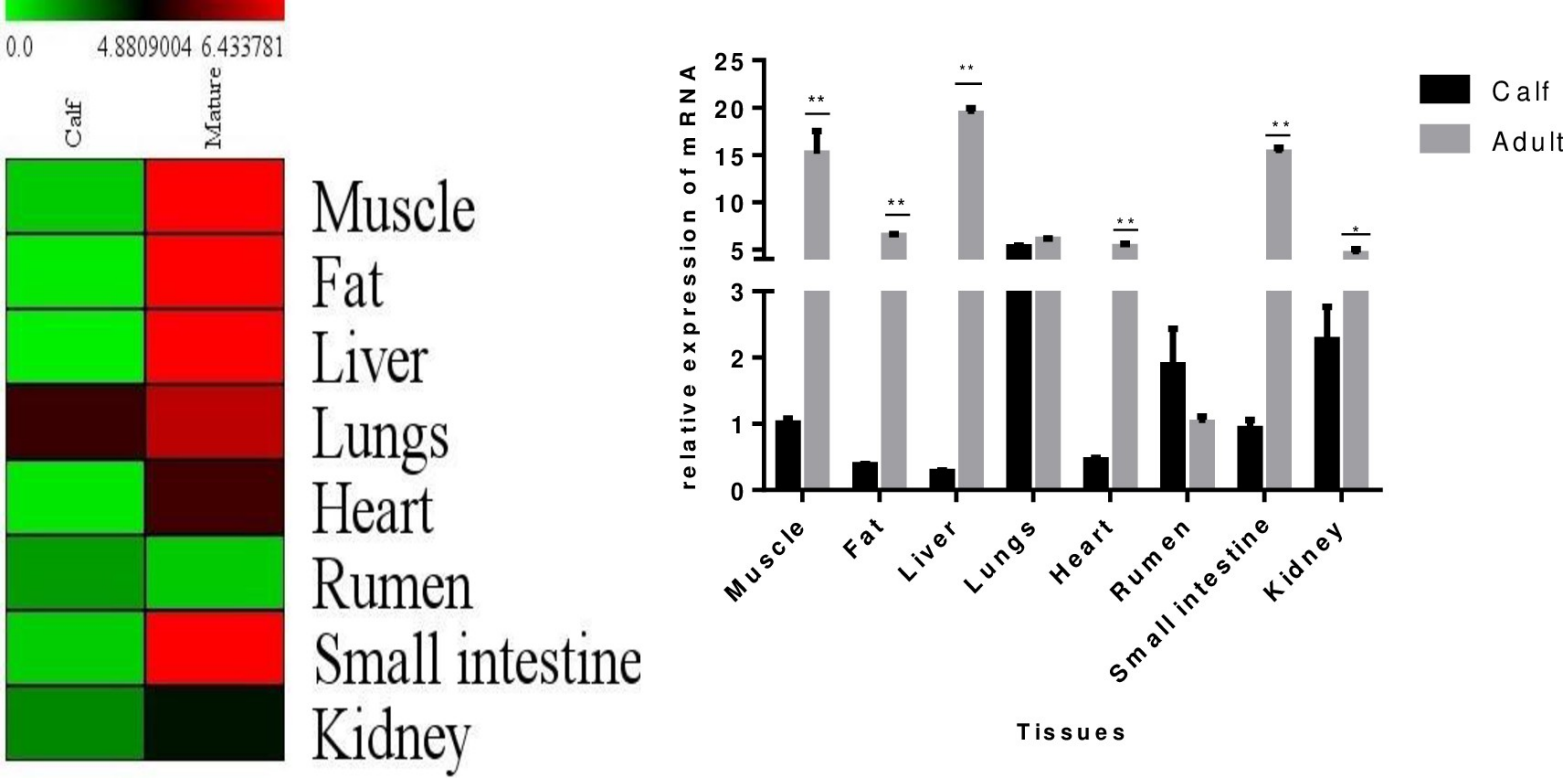
Heat map figure of different tissues mRNA expression level in calf and mature Qinchuan cattle (A), Changes in tissues mRNA expression level with age in Qinchuan cattle (B).

Relative mRNA expression was identified in eight different tissues (muscle, fat, liver, lung, heart, rumen, small intestine, and kidney) and within two different age groups (calf and mature) of Qinchuan cattle (Fig 5A and 5B). In the calf group, the highest mRNA expression was found in lung, followed by kidney, rumen, muscle, small intestine, heart, and fat, and the lowest expression level was found in liver. In the adult age group, the highest expression was found in the liver, muscle, small intestine, fat, lung, heart, kidney and rumen tissues, respectively. The heat map shows that in the calf group, the expression level of the *TORC2* gene was low in all tissues except in the lung, which exhibited moderate expression levels. In mature Qinchuan cattle, the expression levels were high in the small intestine, liver, fat, and muscle; moderate in the lung; and low in the rumen. Significant increases (p<0.01 and p<0.05) in the relative mRNA expression level of *TORC2* in liver, small intestine, muscle, fat, and kidney tissues were found with growth from the calf stage to the adult stage group. No statistically significant variation was found in the expression of the *TORC2* gene in lung and rumen tissues during growth from calf to adult stage. The results obtained in the present study suggest the role of *TORC2* in growth and fat deposition traits in Qinchuan cattle.

## 4. Discussion

The results of the present study showed a significant association of genotypes, haplotypes and diplotypes with body measurement and carcass quality traits. The genotypes GG and TT showed better phenotypes for body measurement and carcass quality traits; similarly, the diplotypes H1-H3 (GG-CC-AT) and H1-H2 (GG-CT-AT) contained the same genotypes GG and AT from SNP1 and SNP3. Moreover, findings of the dual luciferase reporter assay also explored highest transcriptional activity of the haplotype H1 (GCA) in bovine preadipocytes. The probable reason of exhibiting significant association of genotype GG (SNP1), haplotype GCA (H1) with meat quality and body measurement traits; and highest transcriptional activity in bovine preadipocytes may be the gain of transcription factor binding sites due to genotype GG. As in SNP1, genotype GG caused a gain of six new transcription factor binding sites, including *KLP6*, *KLF2*, *Myc zinc finger*, *Myeloid zinc finger 1*, *Heat shock factor 2* and *SPI1*. In SNP2, the substitution of nucleotide C with T caused a gain of only one new TF binding site and a loss of three important TF binding sites. This may be the probable cause of lower body measurement and carcass quality traits shown by the animals with SNP2. Moreover, the logic behind the better phenotypic performance of the individuals possessing genotypes AT in SNP3 may be no alteration in the binding site sequences of the important transcription factors. In silico analysis revealed that SNP3 neither gained nor loss of any new transcription factor binding site in the DNA sequence; however, this locus already contained binding sites for the *MZF1*, *KLF1* and *ZNF263* transcription factors, which remained intact as no loss occurred at this position. Moreover, previously, we confirmed the role of *ZNF263* transcription factor in the regulation of *TORC2* gene as transcriptional activator [10] Furthermore, SNP3 is located in the proximal minimal promoter region; therefore, these transcription factors may have a substantial role in the regulation of the *TORC2* gene. In previous experiments conducted by our research group in the same breed of Qinchuan cattle, we demonstrated the role of the KLF transcription factor family and the *MZF1* transcription factor in the regulation of genes responsible for adipogenesis and myogenesis [45, 46]. The KLFs (Kruppel-like factors) are members of the zinc finger transcription factor group, which binds to the consensus 5’-C(A/T)CCCC-3’ motif in the promoter of various genes that regulates adipogenesis and myogenesis [47–51]. Hence, the role of this location may be due to these transcription factor binding sites in the regulation of the *TORC2* gene. Transcription factor binding sites are the actual controlling factor of gene function. Binding of transcription factors with their respective binding sites at specific locations in the genome regulates gene function [52]. Therefore, genotypes AT and GG at loci SNP3 (g.16535044) and SNP1 (g.16534694), respectively, were the best variants of the *TORC2* gene. Haplotype H1 (GCA) was the most frequent haplotype. The probable cause could be artificial selection in the Qinchuan cattle population, particularly the genomic regions influencing traits of economic importance [53, 54]. Moreover, the evolutionary conservation analysis conducted for 10 species exhibited close homology within the protein sequences of common livestock species, which predicts functional similarity of *TORC2* gene in these target species. In addition, to further exploit the function of the *TORC2* gene in the growth and development of Qinchuan cattle, spatiotemporal mRNA expression was investigated in calf and adult tissues. High expression in the liver is in line with findings of previous studies [6, 55, 56], where its core function is glucagon-mediated activation of hepatic gluconeogenesis [3, 5, 6] to maintain energy balance in vital tissue of the body [57, 58]. Second, a significant (p<0.01) increase in the mRNA expression level of the *TORC2* gene in the small intestine is in line with the findings of Liuqin et al., 2017. They further concluded that the AMP-activated protein kinase (AMPK) pathway, which is regulated by the *TORC2* gene, is mainly responsible for water and ionic homeostasis in the small intestine in pigs [59]. After the small intestine, we found a significant increase in the *TORC2* gene mRNA relative expression in muscle and fat tissue. The role of *TORC2* through the CREB pathway is responsible for skeletal muscle functioning and myogenesis, glucose homeostasis and lipid metabolism in adipocytes [60, 61]. Moreover, the *TORC2* gene regulates adipogenesis and glucose homeostasis and monitors insulin sensitivity [62]. Previous literature has shown that the *TORC2* gene functions as a nutrient transporter and regulates adipogenesis through regulation of the transcriptional activity of *PPARγ* [63]. Various findings [2, 3, 6, 61, 64, 65] confirmed the significant roles of the *TORC2* gene in cell growth, nutrient metabolism, gluconeogenesis, myogenesis and adipogenesis. Therefore, we can conclude that variants in *TORC2* gene might be good markers for body measures and carcass quality traits in the breed improvement program of Qinchuan cattle.

Based upon the findings of the present study, we mapped variants as genotypes GG and AT in SNP1 at locus g.16534694 and SNP3 at locus g.16535044, respectively; haplotypes H1 (GCA) and H4 (ATT); and diplotypes H1-H3 (GG-CC-AT) and H1-H2 (GG-CT-AT) within *TORC2* can be used in marker-assisted selection for body measurement and carcass quality traits in breed improvement programs of Qinchuan cattle.

## Supporting information

S1 Table (XLSX)

S1 Fig TORC2 protein sequences (multiple sequence alignment) of ten species. The level of similarity is shown through background shading of sequence text; the black shades reflect 100%; the grey with black represent 80%; the grey with white shade shows 60%; while white color delineates not conserved.
(DOCX)
