# Supplementary material for: Genetic variants in the TORC2 gene promoter and their association with body measurement and carcass quality traits in Qinchuan cattle
Source: PLoS One. 2020 Feb 14;15(2):e0227254. doi: 10.1371/journal.pone.0227254 (PMC7021310; doi:10.1371/journal.pone.0227254)
Supplement: S1 Fig — The level of similarity is shown through background shading of sequence text; the black shades reflect 100%; the grey with black represent 80%; the grey with white shade shows 60%; while white color delineates not conserved. (DOCX) [file pone.0227254.s002.docx]

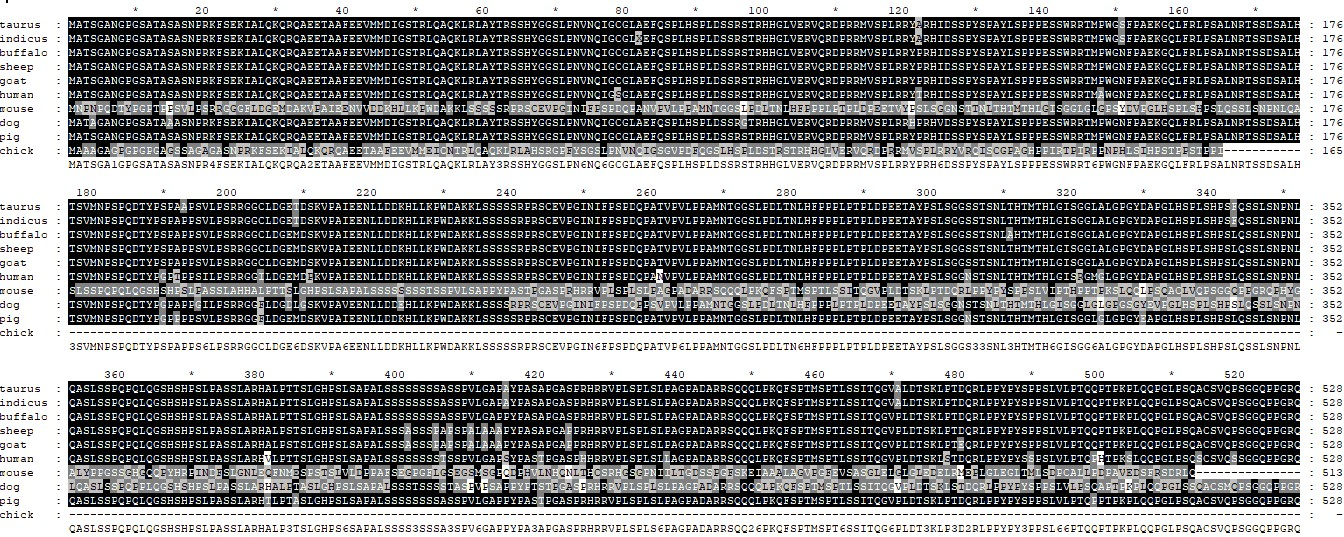

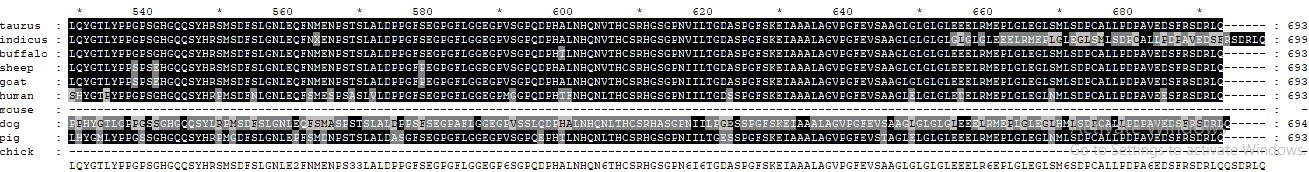


Figure S1. TORC2 Protein Sequences (Multiple sequence alignment) of ten species. The level of similarity is shown through background shading of sequence text; the black shades reflect 100%; the grey with black represent 80%; the grey with white shade shows 60%; while white color delineates not conserved.
